# Impact of Experimental Human Pneumococcal Carriage on Nasopharyngeal Bacterial Densities in Healthy Adults

**DOI:** 10.1371/journal.pone.0098829

**Published:** 2014-06-10

**Authors:** Joshua R. Shak, Amelieke J. H. Cremers, Jenna F. Gritzfeld, Marien I. de Jonge, Peter W. M. Hermans, Jorge E. Vidal, Keith P. Klugman, Stephen B. Gordon

**Affiliations:** 1 Hubert Department of Global Health, Rollins School of Public Health, Emory University, Atlanta, Georgia, United States of America; 2 Laboratory of Pediatric Infectious Diseases, Department of Pediatrics, Radboudumc, Nijmegen, Netherlands; 3 Respiratory Infection Group, Liverpool School of Tropical Medicine, Liverpool, United Kingdom; Public Health England, United Kingdom

## Abstract

Colonization of the nasopharynx by *Streptococcus pneumoniae* is a necessary precursor to pneumococcal diseases that result in morbidity and mortality worldwide. The nasopharynx is also host to other bacterial species, including the common pathogens *Staphylococcus aureus*, *Haemophilus influenzae*, and *Moraxella catarrhalis*. To better understand how these bacteria change in relation to pneumococcal colonization, we used species-specific quantitative PCR to examine bacterial densities in 52 subjects 7 days before, and 2, 7, and 14 days after controlled inoculation of healthy human adults with *S. pneumoniae* serotype 6B. Overall, 33 (63%) of subjects carried *S. pneumoniae* post-inoculation. The baseline presence and density of *S. aureus*, *H. influenzae*, and *M. catarrhalis* were not statistically associated with likelihood of successful pneumococcal colonization at this study’s sample size, although a lower rate of pneumococcal colonization in the presence of *S. aureus* (7/14) was seen compared to that in the presence of *H. influenzae* (12/16). Among subjects colonized with pneumococci, the number also carrying either *H. influenzae* or *S. aureus* fell during the study and at 14 days post-inoculation, the proportion carrying *S. aureus* was significantly lower among those who were colonized with *S. pneumoniae* (*p* = 0.008) compared to non-colonized subjects. These data on bacterial associations are the first to be reported surrounding experimental human pneumococcal colonization and show that co-colonizing effects are likely subtle rather than absolute.

## Introduction

*Streptococcus pneumoniae* (commonly called the pneumococcus) is a resident of the human nasopharynx as well as an important pathogen responsible for 1.3 million deaths in children under 5 annually [1]. Colonization of the nasopharynx is a necessary precursor to pneumococcal diseases including pneumonia, meningitis, and otitis media [2]. *S. pneumoniae* (Sp) shares the nasopharyngeal niche with other bacterial species including the pathogens *Staphylococcus aureus* (Sa), *Haemophilus influenzae* (Hi) and *Moraxella catarrhalis* (Mc). With the introduction of pneumococcal conjugate vaccines (PCV) worldwide, the relationship between nasopharyngeal carriage of Sp and other bacterial pathogens is of special interest. Cross-sectional studies have demonstrated an inverse association between carriage of *S. aureus* and vaccine-type *S. pneumoniae* [3]–[5] and studies of PCV introduction have found increased prevalence of *S. aureus* carriage following PCV roll-out [6]–[8]. Other cross-sectional studies have found positive correlations between Sp and Hi [5], [9], [10], and between Sp and Mc [11]–[13], while a longitudinal study by Spijkerman *et al*. found that the prevalence of Hi increased 3 and 4.5 years after PCV-7 administration but the prevalence of Mc remained unchanged [8]. However, no study to date has examined the ecological effect of controlled inoculation with Sp on other bacterial residents of the nasopharynx.

The Experimental Human Pneumococcal Carriage (EHPC) study [14] is a model of pneumococcal carriage in healthy human adults with the potential for testing vaccine candidates using prevention of carriage as an alternative endpoint. Early results have indicated that Sp challenge provokes mucosal immunity even when Sp carriage is not established [15] and induced carriage offers protection against carriage when re-challenged with the same serotype [16]. In addition to immunological responses produced by experimental carriage, there is also the possibility of altered nasopharyngeal bacterial carriage as a consequence of inter-bacterial competition. We hypothesized that successful colonization with Sp may be affected by the bacteria present in the nasopharynx at baseline and that the addition of pneumococcus to the nasopharynx could alter the carriage of other bacterial pathogens. Using DNA extracted from EHPC nasal wash samples and real-time quantitative PCR (qPCR), we sought to determine whether the presence of Sa, Hi, or Mc prior to inoculation could predict successful Sp colonization and if carriage density of these three bacterial species was altered by successful Sp colonization.

## Methods

### Study Design

Specimen collection and sample processing was conducted in Liverpool, UK, DNA was extracted in Nijmegen, Netherlands, and qPCRs were conducted in Nijmegen and Atlanta, Georgia, USA. Healthy, non-smoking adults between the ages of 18 and 60 were recruited in Liverpool, UK. Exclusion criteria included natural colonization at baseline as determined by traditional culture methods and regular contact with at risk individuals, such as young children. The study was approved by the United Kingdom’s National Research Ethics Service (NRES), Sefton, Liverpool 11/NW/0592 and written informed consent was obtained from all subjects enrolled. Subjects were experimentally colonized with 60, 80, 160, or 320 thousand CFUs of *S. pneumoniae* 6B strain BHN418 [17]. To increase power subjects inoculated with 60 k CFU (n = 22), 80 k CFU (n = 10), 160 k CFU (n = 10) and 320 k CFU (n = 10), were combined into one group of 52 subjects for final analysis.

### Sample Collection

Nasal wash samples were collected as previously described [18], [19] from subjects 7 days before, and 2, 7, and 14 days after inoculation. Directly after collection, 2 ml of a 20 ml nasal wash was mixed and incubated with 4 ml RNAprotect Bacteria Reagent (Qiagen Inc., Valencia CA). It was stored at −80°C until genomic DNA extraction. The remaining volume nasal wash was centrifuged for 10 min at 3345×g. The supernatant was removed and the pellet resuspended in skim milk-tryptone-glucose-glycerol (STGG) medium before plating on blood and chocolate agar plates for identification of Sp, Hi, Sa, and Mc.

### DNA Extraction

The nasal wash RNAprotect mixture was pelleted by centrifugation at 3,345×g for 10 min and the pellet was resuspended in phenol and 300 µl lysis buffer (AGOWA mag Mini DNA Isolation Kit, AGOWA, Berlin, Germany). Then 25–50 mg zirconium beads were added and the sample disrupted by TissueLyser (Qiagen Inc., Valencia CA) for 2 min, twice. The supernatant containing the released DNA was then purified according to the protocol included with the AGOWA mag Mini DNA isolation Kit. Samples were eluted in 63 µl of elution buffer.

### Quantification of Bacterial DNA

When compared to culture-based methods, qPCR has excellent sensitivity and good specificity in detecting *S. pneumoniae* in nasal wash samples and a lower limit of detection (Gritzfeld JF *et al.*, unpublished data). Therefore, we used qPCR to quantify *S. pneumoniae, H. influenzae*, *S. aureus*, and *M. catarrhalis* DNA densities for this study. Reactions were conducted in a volume of 20 µl containing the TaqMan Universal PCR Master Mix (Invitrogen by Life Technology, CA, USA), 1.0 µl of sample DNA, forward and reverse primers and fluorogenic probes (Table 1). For all reactions, the qPCR conditions were 50°C for 2 min, 95°C for 10 min, followed by 40 cycles of 95°C for 15 s and 60°C for 1 min. All samples were run in duplicate; if duplicates mismatched, samples were considered negative if the positive duplicate had Ct>38.

**Table 1:** Primers and probes used for qPCR assays.

Species (gene)	Primer name	5′–3′ nucleotide sequence^a^	Size(bp)	Source
*S. pneumoniae (lyta)*	lytaF	ACGCAATCTAGCAGATGAAGC	101	[31]
	lytaR	TGTTTGGTTGGTTATTCGTGC		
	lytaPr	TTTGCCGAAAACGCTTGATACAGGG		
*S. aureus (nuc)*	nucF	GTTGCTTAGTGTTAACTTTAGTTGTA	154	[32]
	nucR	AATGTCGCAGGTTCTTTATGTAATTT		
	nucPr	AAGTCTAAGTAGCTCAGCAAATGCA		
*H. influenzae (hpd)*	hpdF729	AGATTGGAAAGAAACACAAGAAAAAGA	113	[33]
	hpdR819	CACCATCGGCATATTTAACCACT		
	hpdPr762i	AAACATCCAATCG”T”AATTATAGTTTACCCAATAACCC		
*M. catarrhalis (copB)*	copbF	CGTGTTGACCGTTTTGACTTT	125	[13]
	copbR	TAGATTAGGTTACCGCTGACG		
	copbPr	ACCGACATCAACCCAAGCTTTGG		

a All probes were labeled with Hex at 5′-end and Black Hole Quencher (BHQ) at the 3′-end with the exception for hpdPr762, which was labeled with BHQ at an internal “T” and SpC6 at the 3′-end.

Samples from the dose-ranging study (80 k, 160 k, and 320 k CFU) were analyzed using an Applied Biosystems 7500 Fast Real-Time PCR System and samples from the reproducibility study (60 k CFU) were analyzed on a Bio-Rad CFX96 Real-Time System. Primer and probe concentrations were optimized for each target and each machine. For the ABI 7500, final primer/probe concentrations were 100/100 nM for *lytA*, 200/200 nM for *nuc*, 300/300 nm for *hpd*, and 100/100 nm for *copB*. For the Bio-Rad CFX96, final primer/probe concentrations were 100/100 nM for *lytA*, 400/200 for *nuc*, 300/300 nM for *hpd*, and 200/200 nm for *copB*.

Standard curves were created using purified genomic DNA extracted from laboratory reference strains and quantified using the NanoDrop ND-1000. For *S. pneumoniae,* DNA was extracted from TIGR4 [20]. For *S. aureus,* DNA was extracted from ATCC 29213. For *H. influenzae,* DNA was extracted from R2866 [21]. And for *M. catarrhalis,* DNA was extracted from BBH18 [22].

### Back-calculating Genomes/ml from DNA Quantities

As a measure for bacterial density, we determined the number of bacterial genomes per ml of nasal by back-calculating the number of bacterial genomes based on the bacterial genome size (e.g. for Sp 2160842) and the average weight of a DNA basepair (650 daltons). The number of genome copies per ul of extracted DNA = (mass in ng * Avagadro’s number)/(genome length * 1e9 * 650). This number was then multiplied by 31.5 to account for the difference between the volume of nasal wash used in the extraction (2 ml) and the volume of extracted DNA resulting from the reaction (63 µl) in order to determine genome copies per ml of nasal wash. Any sample with a concentration of >100 genomes/ml was considered to be positive for that bacterial species. Successful colonization was defined as a positive qPCR at any time point following inoculation.

### Statistical Analyses

Given the non-normal distribution of the data (as assessed by the Shapiro-Wilk test) numbers are reported as median (interquartile range) except when noted. Differences in proportions were assessed by the Fisher exact test and differences in continuous variables (such as density) were assessed using the Mann-Whitney U test. An α = 0.05 was considered statistically significant for all analyses. When assessing difference in density of multiple groups, a Kruskall-Wallis analysis of variance was employed. G*Power 3.1 [23] software was used for power calculations and all other statistical analyses were conducted in GraphPad Prism 6 or RStudio 0.97.

## Results

### Sp Inoculation Induces Pneumococcal Carriage as Assessed by qPCR

**Figure 1:**
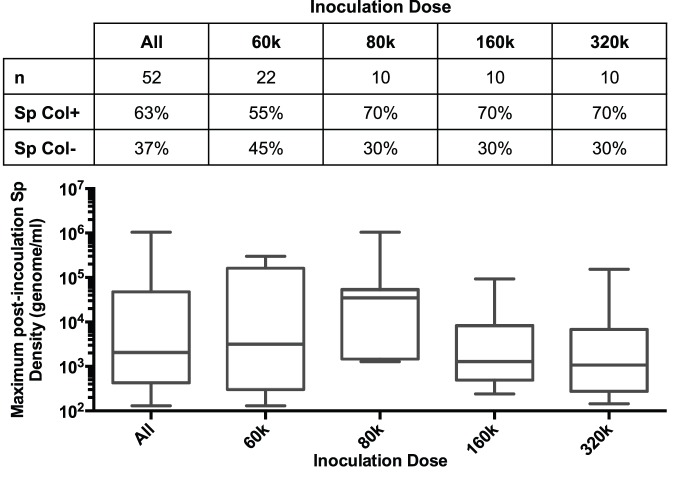
Colonization rates and maximum post-inoculation densities at four inoculation doses. Subjects were administered intra-nasal doses of 60 k, 80 k, 160 k, or 320 k CFU of *S. pneumoniae* 6B. Comparison of Sp colonization rates and maximum post-inoculation densities rates among subjects in the four dose groups revealed no statistically significant differences. Boxplots indicate median, interquartile range, and range with circles indicating outlier values.

Of the 52 subjects inoculated, 33 individuals (63%) were Sp colonized post-inoculation. Subjects receiving different inoculation doses of Sp were grouped together to increase the sample size of this analysis. To verify that all subjects were experiencing biologically similar challenges, we compared the frequency and density of pneumococcal colonization among these groups. Of the subjects in the 60 k CFU group, 55% became colonized with Sp while 70% of subjects in the 80 k, 160 k, and 320 k groups became colonized (Figure 1). The maximum post-inoculation colonization log densities (genomes/ml) of the 60 k, 80 k, 160 k, and 320 k CFU groups were 3.5 (2.5–5.0), 4.5 (3.7–4.7), 3.1 (2.9–3.8), and 3.0 (2.7–3.5), respectively (Figure 1). There was no statistically significant difference between the four groups in maximum density (p = 0.30) or colonization rate (p = 0.47).

### Carriage Rates and Densities over Time

**Figure 2:**
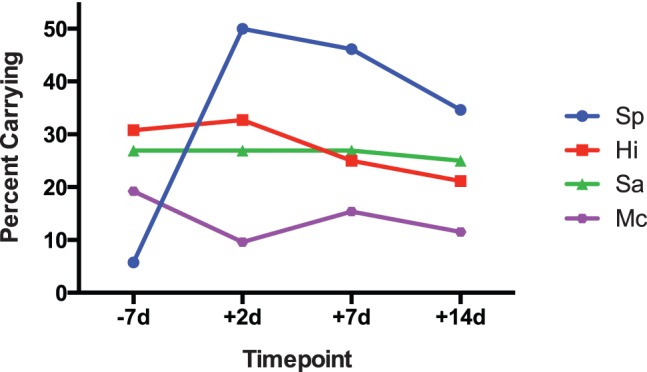
Percent of subjects carrying Sp, Hi, Sa, and Mc at baseline and at 2, 7, and 14 days post-inoculation. Nasopharyngeal samples from 52 subjects were examined at four time points for bacterial DNA and carriage was defined as >100 genomes/ml.

While all subjects were culture negative for *S. pneumoniae* at baseline, 3 subjects (6%) were Sp carriage positive at baseline as assessed by qPCR. Following inoculation, carriage of Sp increased to 50% at 2 days post-inoculation and then fell to 46% and 34% at 7 and 14 days post-inoculation, respectively (blue line, Figure 2.

At baseline, 16 subjects (31%) carried Hi, 14 subjects (27%) carried Sa, and 10 subjects (19%) carried Mc (Figure 2). Hi was carried by 33%, 25% and 21% of subjects 2, 4, and 7 days post-inoculation, respectively (red line, Figure 2). Sa was carried by 27%, 27% and 25% of subjects 2, 4, and 7 days post-inoculation, respectively (green line, Figure 2). Mc was carried by 10%, 15%, and 12% of subjects 2, 4, and 7 days post-inoculation, respectively (purple line, Figure 2). Overall, while the rate of Sp carriage increased dramatically after inoculation, the carriage rates of Sa, Hi, and Mc did not significantly change.

**Figure 3:**
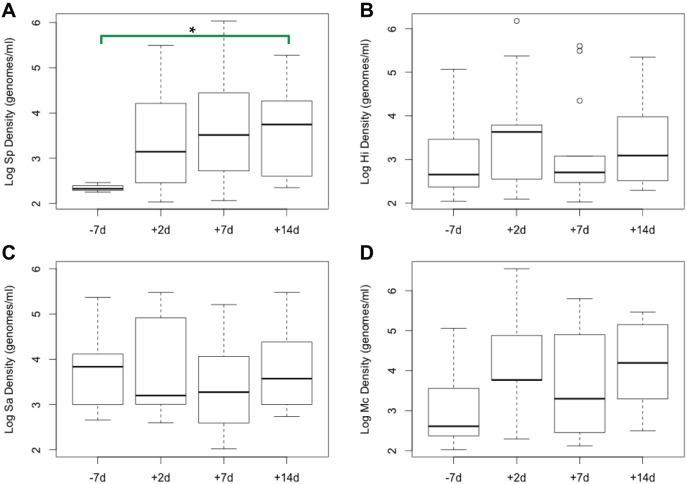
Density of Sp (A), Hi (B), Sa (C), and Mc (D) in nasal wash samples at four time points. Boxplots of log bacterial density (in genomes/ml) of all samples with >100 genomes/ml at each time point. Boxplots indicate median, interquartile range, and range with circles indicating outlier values. Asterisk indicates *p* = 0.003.

When carriage densities were examined for each bacterium over the four time points, we observed an increase in the median log density of Sp; rising from 2.3 at baseline to 3.7 at 14 days post-inoculation (*p* = 0.003; Figure 3A). There was no significant change over time in the density of *H. influenzae* (Figure 3B), *S. aureus* (Figure 3C), or *M. catarrhalis* (Figure 3D).

### Baseline Bacterial Carriage as a Predictor of Sp Colonization

To assess whether carriage of Hi, Sa, or Mc was associated with likelihood of Sp colonization post-inoculation, we examined carriage as both a binary variable (detected) and a continuous variable (density). While 75% of those Hi+ at baseline would go on to become colonized with Sp, only 58% of those Hi− would become Sp colonized (*p* = 0.71; Table 2). Conversely, 50% of Sa+ became colonized with Sp while 68% of Sa− became colonized with Sp (*p* = 0.33; Table 2). Individuals carrying Mc at baseline became colonized with Sp 50% of the time while individuals not carrying Mc at baseline became colonized with Sp 57% of the time (*p* = 0.47; Table 2). Similarly, Mann-Whitney U tests revealed no statistically significant difference in baseline densities of Mc, Sa, or Hi when comparing those colonized with Sp to those not colonized by Sp (data not shown).

**Table 2:** Contingency tables of carriage of Hi, Sa, and Mc at baseline against colonization with Sp post-inoculation for 52 subjects.

Carriage at baseline	Sp Col+(n = 33)	Sp Col−(n = 19)	Fisher exact p-value
Hi+ (n = 16)	12 (75%)	4 (25%)	0.71
Hi− (n = 36)	21 (58%)	15 (42%)	
Sa+ (n = 14)	7 (50%)	7 (50%)	0.33
Sa− (n = 38)	26 (68%)	12 (32%)	
Mc+ (n = 10)	5 (50%)	5 (50%)	0.47
Mc− (n = 42)	28 (67%)	14 (33%)	

### Hi, Sa, and Mc Carriage Post-inoculation

**Figure 4:**
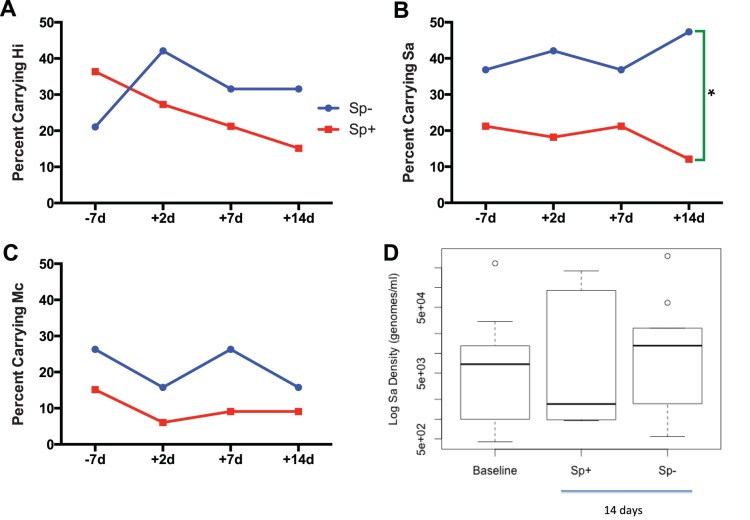
Percent carrying Hi (A), Sa (B), and Mc (C) at 4 time points and density of Sa colonization at baseline and 14 days (D) segregated by success of pneumococcal colonization. Numbers of subjects carrying each bacteria ranged from 11**–**17 for *H. influenzae*, 5**–**10 for *M. catarrhalis*, and 13**–**14 for *S. aureus*. Asterisk indicates *p* = 0.008.

While the proportion of subjects carrying Hi decreased from 31% pre-inoculation to 21% post-inoculation (Figure 2), to determine if there was a change in Hi related to successful Sp colonization, we segregated the data by Sp colonization status. Among those colonized by Sp, carriage of Hi decreased from 36% pre-inoculation to 15% 2 days post-inoculation (red line; Figure 4A). Among those not colonized by Sp, carriage of Hi increased from 21% pre-inoculation to 42% 2 days post-inoculation (blue line; Figure 4A). However, the difference in proportions carrying Hi between the Sp colonized group and the Sp uncolonized group was not statistically significant at any of the four time points measured.

For *S. aureus*, 37% of the Sp uncolonized group carried Sa at baseline, while 21% of the Sp-colonized group carried Sa at baseline; this difference in proportions was not statistically significant (*p* = 0.33). Similarly the carriage rates of Sa were not significantly different between the two groups at 2 and 7 days post-inoculation. However, at 14 days post inoculation, those colonized with Sp had a significantly lower rate of Sa carriage (12%) than those uncolonized with Sp (47%; *p* = 0.008; Figure 4B).

Among those colonized by Sp, carriage of Mc decreased from 15% pre-inoculation to 9% post-inoculation (red line; Figure 4C). Among those not colonized by Sp, carriage of Mc decreased from 26% pre-inoculation to 16% post-inoculation (blue line; Figure 4C). The difference in proportions between the Sp colonized group and the Sp uncolonized group was not statistically significant at any of the four time points.

To further characterize the difference in Sa carriage at 14 days post-inoculation, we compared the density of Sa carriage among those colonized by Sp to those uncolonized by Sp. Among those carrying Sa at this time point, the median log density was lower among those colonized by Sp (3.2) than those uncolonized by Sp (4.1; Figure 4D); however this difference was not statistically significant (*p* = 0.71).

## Discussion

This is the first study to examine bacterial densities of *S. aureus*, *H. influenzae*, and *M. catarrhalis* before and after controlled addition of *S. pneumoniae* to the human nasopharynx. Serotype 6B is a clinically important serotype of *S. pneumoniae*; a recent meta-analysis found that pneumonia patients infected with 6B were at an increased risk of death [24]. The prevalence of 6B in carriage and disease led to its inclusion in all formulations of the pneumococcal conjugate vaccine. While a smaller, previous study examined the immune response to experimental human pneumococcal carriage, that study did not characterize the bacterial load in the nasopharynx before and after inoculation [25]. Therefore, the current study of pneumococcal colonization of healthy adults offers the first chance to estimate effect size for two separate questions. First: do baseline bacterial densities correlate with likelihood of Sp colonization? And second: does successful pneumococcal colonization lead to a change in the presence or density of other nasopharyngeal species?

On examining 52 subjects, 33 (63%) of whom were successfully colonized by Sp, we found no statistically significant association between baseline presence or density of Hi, Sa, or Mc and likelihood of colonization with Sp. However, there were tendencies in the data which suggest that successful pneumococcal colonization may be more likely in those carrying Hi at baseline and less likely in those carrying Sa; these are associations consistent with previous studies [3], [4], [6], [26]–[28]. While the current study only had a power of 0.22 to detect a difference in proportions of 17% (i.e. the difference in percent colonized by Sp when segregating by Hi carriage status), a future study could enroll approximately 250 subjects to achieve a power of 0.8 now that the estimated effect size is better described.

Successful colonization with Sp was associated with an absence of Sa in samples 14 days post-inoculation. This difference in proportions was due to both a decrease in prevalence of Sa in subjects colonized with Sp and an increase in prevalence of Sa in those uncolonized with Sp. The increase in Sa prevalence may have been incidental or it may have been an immunologically-mediated phenomena related to failed pneumococcal colonization. While these data are not sufficient to prove that colonization with Sp always decreases prevalence of Sa at 14 days post-colonization, a time-delayed effect on *S. aureus* carriage may implicate an immunologic mechanism in the antagonistic relationship between Sp and Sa. Notably, studies of HIV-positive children have demonstrated a lack of antagonism between Sa and Sp [6], [29] and a recent study has demonstrated that pneumococcal colonization in mice elicits cross-reactive igG antibodies that protect against *S. aureus* [30]. Future studies may want to measure levels of the cross-reactive antibodies, staphylococcal P5CDH and pneumococcal SP_1119, identified by Lijek *et al*. However, the difference in proportions we observed at 14 days was an exacerbation of a pre-existing difference in proportions at baseline, 2 and 7 days post-inoculation. Therefore, repeated studies are needed to verify this observed delayed effect on Sa carriage.

This study did not show a statistically significant difference in prevalence or density of *H. influenzae* and *M. catarrhalis* following controlled inoculation with *S. pneumoniae*. While the prevalence of *M. catarrhalis* decreased from baseline to 2 days post-inoculation, this change did not meet criteria for statistical significance and the decrease in prevalence was seen equally in subjects colonized by Sp and subjects not colonized with Sp. Either controlled Sp colonization of healthy adults does not change prevalence and density of Hi and Mc, or this study was not adequately powered to detect the magnitude of change present.

The primary limitations of this study include sample size and the lack of sham-inoculated controls. *A priori*, it was difficult to estimate the effect size of bacterial carriage on pneumococcal colonization and *vice versa*. From this study, it appears that the effect size of baseline carriage of Sa and Hi on likelihood of pneumococcal colonization is in the range of 15–20%. Similarly, the examination of changes in bacterial carriage post-inoculation was limited by the small number of subjects carrying Hi, Sa, or Mc; with only 19–31% of subjects carrying these microbes, the comparison of those Sp-colonized to those uncolonized with Sp is very limited. Future studies may want to examine a population in which carriage of these bacterial species is more prevalent. While this study examined bacterial population dynamics in adults, colonization with Sp and other nasopharyngeal pathogens is most common in children; however, ethical considerations may preclude experimental pneumococcal challenge studies from being conducted in a pediatric population. Finally, the design of this study enabled the comparison of successful colonization with unsuccessful colonization without examining the effect of repeated nasal washes on nasopharyngeal bacterial population dynamics. While nasal washes are an effective way to determine nasopharyngeal carriage [18], it is unknown how this procedure may disturb the nasopharyngeal microbial community.

This study is the first to examine nasopharyngeal bacterial densities in healthy human adults inoculated with Sp. While children are the primary reservoirs of pneumococcal carriage, experimental studies in adults will likely be the best approximation we can achieve for observing pneumococcal colonization in a controlled environment. The difference we found in prevalence of Sa carriage 14 days post-pneumococcal colonization is an intriguing finding that lends support to the hypothesis that Sp and Sa antagonism is mediated by the host immune system. Larger, future studies will be able to examine the relationship between pneumococcal colonization and Sa, Hi, and Mc carriage, and microbiome studies using 16S sequencing data may reveal associations with species that are unculturable or are not known pathogens. Furthermore, the experimental human pneumococcal carriage model offers future opportunities to examine vaccine efficacy and observe the changes in nasopharyngeal microbiology that occur near the time of vaccination.
